# The complete mitochondrial genome of *Eriocampa ovata* Linné, 1760 (Hymenoptera: Tenthredinidae) and phylogenetic analysis

**DOI:** 10.1080/23802359.2022.2128915

**Published:** 2022-10-12

**Authors:** Mengmeng Liu, Min Li, Meicai Wei, Zejian Li

**Affiliations:** a College of Ecology, Lishui University, Lishui, China; b College of Life Sciences, Jiangxi Normal University, Nanchang, China; c Scientific Research and Management Center of East China Medicinal Botanical Garden, Lishui Forestry Bureau, Lishui, China

**Keywords:** Mitochondrial genome, phylogenetic analysis, Tenthredinidae

## Abstract

*Eriocampa* Hartig, 1837 is a small genus of Tenthredinidae and its systematic position has never been fully assessed. The complete mitochondrial genome of *Eriocampa ovata* Linné, 1760 was described. The circular genome is 16,293 bp in length with an A + T content of 80.6%. It contains 37 genes and a 1254 bp control region with a 405 bp repetitive sequence. All the 13 protein-coding genes initiate with a typical ATN. The CR - *trnI* (+)- *trnQ* (−)- *trnM*(+) cluster rearranges to *trnQ* (−)- *trnM* (+)- CR *-trnI*(+). Phylogenetic analysis demonstrates that *E. ovata* and *Conaspidia wangi* Wei, 2015 are closely related within the subfamily of Tenthredinidae.

*Eriocampa* Hartig, 1837 is a small Holarctic genus of Tenthredinidae and mainly occurs in eastern Asia. Its systematic position has never been fully assessed. Ashmead (1898) placed *Eriocampa* into the subfamily Selandriinae of his Selandriidae and thought *Eriocampa* is close to *Caliroa* Costa, and the genus now is a member of Heterarthrinae (Abe and Smith 1991; Taeger et al. 2010) or Blennocampinae (Benson 1952) of Tenthredinidae, or Caliroinae of Heterarthridae (Wei and Nie, 1998), etc. Rohwer (1911) erected Eriocampini for the genus only under Allantinae of Tenthredinidae. Ross (1937) placed *Eriocampa* with *Pseudosiobla* and *Dimorphopterx* together into Eriocampini of Allantinae within the family Tenthredinidae, but he stated that the three genera were grouped together chiefly on the rugose mesopleurae, but they differ radically from each other in mandibular, antennal structure, and genitalia. Benson (1952) thought that Eriocampini included only *Eriocampa* and placed the tribe into his complicated Blennocampinae, which also includes Athaliini, Allantini, Empriini, Fenusini, and Blennocampini. Takeuchi (1952) placed *Eriocampa* with *Eriocampopsis* into Eriocampini under the subfamily Allantinae, which includes Athaliini, Belesini, Empriini, and Allantini; besides Eriocampini. Zombori (1981) placed *Eriocampa* into Eriocampini under a heterogenous subfamily Sellandriinae, which includes 13 tribes, such as Aneugmenini, Athaliini, Empriini, Heptamelini, Heterarthrini, Hoplocampini, etc. Abe and Smith (1991) and Taeger et al. (2010) placed *Eriocampa* into Allantinae without tribal arrangement. Wei and Nie (1998) grouped *Eriocampa* with *Pseudosiobla*, *Eriocampopsis*, *Dimorphopteryx*, and *Armitarsus* into Eriocampini and placed the tribe into Tenthredininae.

Comparing the above systems concerning *Eriocampa*, most of them regarded *Eriocampa* to be a member of Eriocampini and thought that it was close to Allantinae or even placing the genus directly into Allantinae, except for Wei and Nie (1998) thought that *Eriocampa* was not close to Allantinae but a member of Tenthredininae. In this study, we sequenced the mitochondrial genome of *Eriocampa ovata* Linné, 1760 to determine the phylogenetic position of *Eriocampa*.

Specimens of *E. ovata* were collected from Villa Luganese (46.061 N, 9.025 E), Switzerland. Samples were identified by Wei Meicai (weimc@126.com) at the Asia Sawfly Museum, Nanchang (ASMN), where a voucher specimen (CSCS-Hym-MC0143) is kept. Genomic DNA was extracted from a female and sequenced using Illumina Hiseq 4000 platform by following the standard protocols. A total of 12.92 Gb raw data was yielded and used for subsequent genome assembly by MitoZ (Meng et al., 2019) and Geneious Prime 2019.2.1 (http://www.geneious.com). Genome annotation referred to the results produced by MITOS (Bernt et al. 2013), in which the starting and ending of PCGs were determined according to the results of comparative genomics. Each PCG was aligned individually with the MAFFT algorithm in the TranslatorX server (Abascal et al. 2010). The maximum likelihood method was constructed by IQTREE (Nguyen et al. 2015) with the GTR + CAT model and Bayesian inference conducted by PhyloBayes MPI on XSEDE (Lartillot et al. 2009) was used for 13 PCGs and nine unsaturated PCGs, respectively.

The sequence yield by MitoZ was 12,065 bp long. We thoroughly checked the obtained whole sequence by assembling using *Dimorphopteryx* sp. (new species, unpublished) as reference sequences (mean coverage was 18,525). The 1254-length control region (CR) was identified by extending both ends of the above sequence. The circular genome is 16,293 bp long and the overall A + T content is 80.60%. Compared with the ancestral insect mitochondrial genome (Boore 1999), CR-trnI (+)-trnQ (−)-trnM (+) is rearranged to trnQ (−)- trnM (+)-CR-trnI(+). However, within this region, there is only a 9-bp intergenic sequence between trnI and nad2. The other 192 intergenic nucleotides are distributed between 19 gene pairs, with the longest being up to 41 bp between cob and trnS. All 13 PCGs utilize ATN as the start codon, while 9 PCGs end with the canonical triplet stop codon. cob and nad4l use TAG, while nad1 and nad4 end with a single T.

Phylogenetic trees constructed under four strategies constantly support the sister group relationship between *E. ovata* and *Conaspidia wangi* Wei, 2015 (Qi et al., 2015). None of the results support *E. ovata* and *Dimorphopteryx* sp. originating from the same subfamily clade. It confirms the suspicion based on morphological evidence (Ross, 1937). Three results support Selandriinae, rather than Nematinae, as the sister group of *E. ovata* + *C. wangi*. However, Selandriinae, as the most basal branch, is supported by three of the four trees. However, these ununified phylogenetic relationships all indicate that the current sampling is far from enough to solve the internal relations in Tenthredinidae (Figure 1).

**Figure 1.**
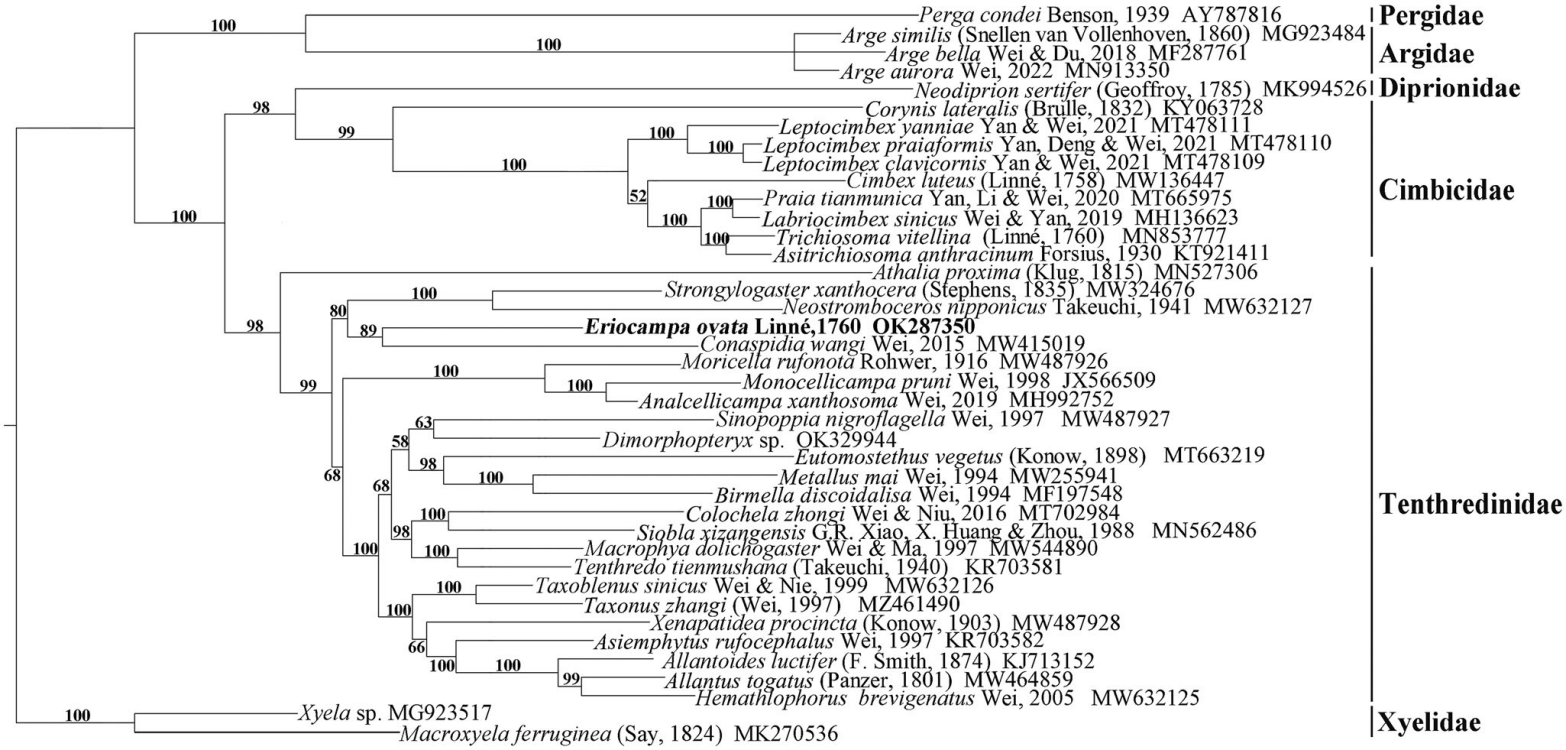
PhyloBayes MPI with the GTR + CAT model on 13 protein-coding sequences of 40 species. Numbers at the left of nodes are bootstrap support values. The accession number of each species is indicated after the Latin name.

## Data Availability

The genome sequence data that support the findings of this study are openly available in GenBank of NCBI at [https://www.ncbi.nlm.nih.gov] (https://www.ncbi.nlm.nih.gov/) under the accession number OK287350. The associated BioProject, SRA, and Bio-Sample numbers are PRJNA764375, SRR15990418, and SAMN21501672, respectively. All related files are publicly available in figshare (https://figshare.com/account/home#/projects/123754).
